# Patient perceptions of in‐hospital laboratory blood testing: A patient‐oriented and patient co‐designed qualitative study

**DOI:** 10.1111/hex.13880

**Published:** 2023-09-26

**Authors:** Surakshya Pokharel, Zoha Khawaja, Jonathan Williams, Adnan Adil Mithwani, Kimberly Strain, Prachi Khanna, Anna Rychtera, Veronika Kiryanova, Karen Tang, Pamela Mathura, Chris Hylton, Anshula Ambasta

**Affiliations:** ^1^ Ward of the 21st Century, Calgary Zone of Alberta Health Services University of Calgary Calgary Canada; ^2^ Department of Medicine, Cumming School of Medicine University of Calgary Calgary Canada; ^3^ Strategy for Patient Oriented Research (SPOR) Support Unit Calgary Alberta Canada; ^4^ Department of Biology, Faculty of Science University of British Columbia Vancouver Canada; ^5^ Strategy for Patient Oriented Research (SPOR) Support Unit Vancouver British Columbia Canada; ^6^ Michael Smith Health Research Vancouver British Columbia Canada; ^7^ O'Brien Institute of Public Health, Cumming School of Medicine University of Calgary Calgary Canada; ^8^ Department of Medicine University of Alberta Edmonton Canada; ^9^ Graduate Studies Laval University Quebec City Canada; ^10^ Department of Anesthesia, Pharmacology and Therapeutics, Therapeutics Initiative University of British Columbia Vancouver Canada

**Keywords:** blood testing in hospitals, laboratory testing in hospitals, patient experience, patient‐oriented research

## Abstract

**Background:**

Indiscriminate use of laboratory blood testing in hospitals contributes to patient discomfort and healthcare waste. Patient engagement in low‐value healthcare can help reduce overuse. Understanding patient experience is necessary to identify opportunities to improve patient engagement with in‐hospital laboratory testing.

**Objectives:**

To understand patient experience with the process of in‐hospital laboratory blood testing.

**Methods:**

We used a qualitative study design via semistructured interviews conducted online or over the phone. Participants were adult patients or family members/caregivers (≥18 years of age) with a recent (within 12 months of interview) experience of hospitalization in Alberta or British Columbia, Canada. We identified participants through convenience sampling and conducted interviews between May 2021 and June 2022. We analysed transcripts using thematic content analysis. Recruitment was continued until code saturation was reached.

**Results:**

We interviewed 16 participants (13 patients, 1 family member and 2 caregivers). We identified four themes from patients' experiences of in‐hospital laboratory blood testing: (i) patients need information from healthcare teams about expected blood testing processes, (ii) blood draw processes should consider patient comfort and preferences, (iii) patients want information from their healthcare teams about the rationale and frequency of blood testing and (iv) patients need information on how their testing results affect their medical care.

**Conclusion:**

Current laboratory testing processes in hospitals do not facilitate shared decision‐making and patient engagement. Patient engagement with laboratory testing in hospitals requires an empathetic healthcare team that provides clear communication regarding testing procedures, rationale and results, while considering patient preferences and offering opportunities for involvement.

**Patient or Public Contribution:**

We interviewed 16 patients and/or family members/caregivers regarding their in‐hospital laboratory blood testing experiences. Our findings show correlations between patient needs and patient recommendations to make testing processes more patient‐centred. To bring a lived‐experience lens to this study, we formed a Patient Advisory Council with 9–11 patient research partners. Our patient research partners informed the research design, co‐developed participant recruitment strategies, co‐conducted data collection and informed the data analysis. Some of our patient research partners are co‐authors of this manuscript.

## 1 INTRODUCTION

Patients value testing as part of their medical care,^1^, ^2^, ^3^ and patient expectation is an often‐cited barrier to reducing low‐value healthcare use.^4^ Laboratory blood testing overuse is one such problem of the healthcare system that wastes resources^5^, ^6^, ^7^ and contributes to patient harm in the form of discomfort from painful sampling, disrupted sleep patterns and hospital‐acquired anaemia, which, in turn, increases the need for blood transfusions, prolongs hospitalization stays and increases mortality.^8^, ^9^, ^10^, ^11^, ^12^, ^13^ Given the direct negative consequences of overutilization of laboratory blood testing on patients, it is crucial to collaborate with patients to understand their experiences and identify opportunities to improve patient engagement. When patients are engaged in their care and are appropriately informed about risks and benefits by their healthcare team, they are likely to choose the optimal use of healthcare resources.^14^, ^15^ A meta‐analysis demonstrated a significant reduction in low‐value care with initiatives that engage patients through patient‐targeted educational materials and/or shared decision‐making tools.^16^ Furthermore, factors such as direct release of information like blood test results to patients,^17^ easy access to online portals^18^, ^19^ and strong physician‐patient relationship^20^, ^21^ have been reported to increase patient engagement and utilization of care. A survey study aimed to understand in‐hospital blood testing from the patient perspective indicated that patients value shared decision‐making and comprehensive communication with regard to their blood tests, but an intervention is needed to provide practical approaches for this to happen.^22^

Despite literature documenting the negative impacts of overutilization of laboratory blood testing in hospitals on patients and the value of patient engagement in reducing such low‐value care, very little is known about these experiences from the patient's perspective. There is a need to improve patient engagement with in‐hospital laboratory blood testing and one way this can be enhanced is by understanding the experiences of the patients. To address this knowledge gap and to generate recommendations for how to improve the process of in‐hospital laboratory testing, we aimed to seek the perspectives of a diverse group of patients, a term that includes ‘individuals with personal experience of a health issue and informal caregivers, including family and friends’ as defined by the Canadian Institutes of Health Research (CIHR).^23^ The goal of this study was to use in‐depth interviews with patients/families/caregivers to understand their experience with in‐hospital blood testing and capture their recommendations to facilitate patient engagement with laboratory testing processes. Findings from this study will help identify how current laboratory testing processes intersect with patient expectations, categorize deficiencies and develop novel embedded educational initiatives to engage patients with in‐hospital laboratory testing.

## 2 METHODS

### 2.1 Patient Advisory Council (PAC)

**Figure 1:**
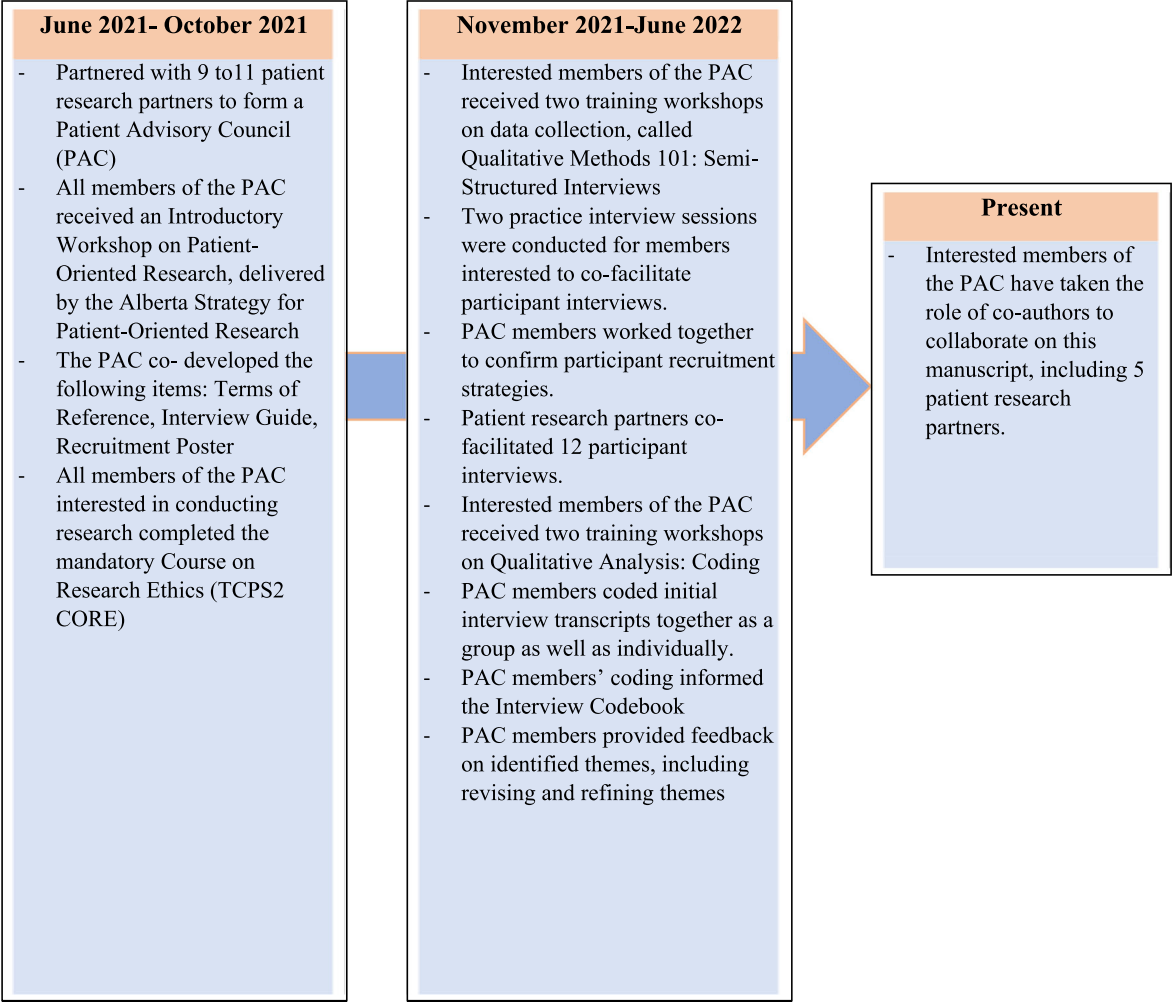
Collaboration with patient research partners through a Patient Advisory Council over the study period.

To bring in a lived‐experience lens to this study, a PAC was formed. This PAC was guided by the CIHR ‘Strategy for Patient‐Oriented Research (SPOR)^’^—Patient Engagement Framework,^23^ which defines patient engagement as activities that happen when patients collaborate as active and equal partners on health research projects that impact them.^23^, ^24^ With support from the Alberta SPOR SUPPORT Unit (AbSPORU),^25^ members of this PAC included  the academic research team, 9–11 patient research partners (some left early on due to competing priorities), and support members from the AbSPORU^25^ Patient Engagement Team. Through the PAC, patient research partners and academic researchers collaborated to develop the study design, data collection and interpretation techniques and dissemination plan (Figure 1). The PAC members were instrumental in: A. co‐developing study materials (e.g., interview guide), B. co‐developing recruitment strategies, C. offering guidance on the scope of the study to ensure that the task of the study was easy for patient participants to understand, D. facilitating participant interviews, E. informing analysis, F. co‐developing scientific publications to disseminate findings and G. assuring usage of culturally appropriate language.

Collaborative work occurred through monthly PAC meetings. Outside of these meetings, patient research partners were offered research‐oriented training workshops on conducting interviews and data analysis. Training workshops were organized and facilitated either by a member of the AbSPORU Patient Engagement Team or academic researchers. Nearly 6 to 8 patient research partners attended each workshop. Patient research partners were compensated for their time and contribution as per CIHR SPOR—Patient Engagement Framework.^23^

### 2.2 Study design

We conducted a multihospital qualitative study between May 2021 to June 2022 across Alberta (AB) and British Columbia (BC) in Canada using convenience sampling^26^ to recruit participants. Recruitment posters were widely distributed online and were displayed in waiting areas of two hospitals, that is, Foothills Medical Centre, AB and St. Paul's Hospital, BC. Eligibility criteria included age ≥18 years and experience with hospitalization (personal or in the role of family/caregiver) in an AB or BC hospital within the preceding 12 months. All participants were offered a $25 gift card. Recruitment was continued until code saturation^27^ was reached, that is, when the coding team could not identify any additional codes. The Consolidated Criteria for Reporting Qualitative Research^28^ guidelines were followed to provide rigour to the study (Supporting Information: Appendix 1).

### 2.3 Data collection

Owing to the COVID‐19 pandemic, all individual semistructured interviews were conducted over the phone or online via Zoom^29^ and were audio‐recorded. Interviews were conducted by 2–3 PAC members, including female academic researchers S. P., Z. K. and A. A., male academic researchers J. W. and A. A. A. and female patient research partners K. S., P. K., A. R. and V. K. All interviewers were provided with training on conducting interviews. Z. K., with training and experience in qualitative research, was present during all online interviews and took field notes. An academic researcher obtained consent from each participant either through email before the interview, or during the initial portion of the interview. Interviewers began each interview by introducing themselves, their role on the research team and their interest in the research topic, as well as the research aims and reasons for conducting the study. The Interview Guide (Supporting Information: Appendix 2) was informed by the interview questions by Roulston^30^ adapted from Patton.^31^ It described the process of laboratory blood testing in hospitals and asked questions regarding participants' experience and suggestions for each stage of the process (i.e., the decision to perform a test, the process of drawing blood, analysis and sharing of test results and use of results to guide care). The interview guide was pilot‐tested within the academic research team and through interview workshops with patient research partners.

### 2.4 Data analysis

Interviews were transcribed, anonymized and verified for accuracy with the audio recordings. Interview transcripts remained within the research team and were not returned to participants for comment and/or correction. We used NVivo12^32^ to analyse the transcripts concurrently with data collection. We followed Braun and Clark's^33^ six‐phase guide to thematic analysis: a. familiarization, b. generating initial codes, c. identifying themes, d. reviewing themes, e. defining themes and f. discussing the write‐up. To begin, academic researchers immersed themselves in the transcribed data and reviewed them individually. Then, to generate initial codes, four transcripts were coded by patient research partners and academic researchers either individually or in duplicate. An Interview Codebook (Supporting Information: Appendix 3) was iteratively developed through this process. This codebook was divided into the same two categories as the interview guide for easy comparison of data, that is, 1. Patient Hospital Experience and 2. Patient Suggestions and Recommendations. Academic researchers Z. K. and S. P. used and applied this codebook in duplicate to the initial four transcripts and to all ongoing interviews, while searching for additional codes. S. P. and Z. K. met weekly to discuss their coding and areas of discrepancies. Academic researcher A. A. was consulted when discrepancies could not be resolved. Theming was not a linear process. S. P. and Z. K. initially identified themes across codes and organized them in tables with verbatim quotes from participants. A. A. and S. P. then analysed the overall coded data separately and further delved into identifying and organizing themes: themes were collapsed together, split into two or more, and even discarded altogether. Academic researchers K. T. and P. M. with qualitative research experience provided revisions on the organization of themes. Themes were then shared with patient research partners (including coauthors K. S., P. K., A. R., V. K. and C. H.) who provided their insight into wording and organization. The themes were then iteratively developed and defined by A. A. and S. P. Finally, A. A. and S. P. discussed how the reporting of these themes should provide a clear association between patients' hospital experiences and suggestions for improvements.

## 3 RESULTS

**Figure 2:**
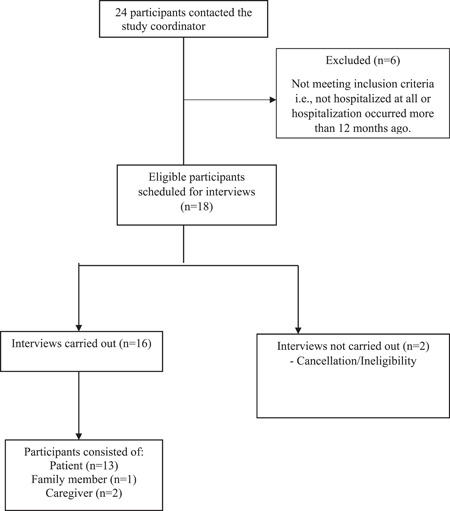
Flow diagram of participant selection for interviews.

Of the 24 interested patient participants who contacted the study coordinator, 16 participants were interviewed based on eligibility and availability (Figure 2). The 16 participant comprised 13 patients, 1 family member and 2 caregivers. Participants represented both provinces, different ages and varied medical comorbidity status (Table 1). Eleven out of 16 participants identified as women. The mean duration of the interviews was 41 min (range: 22–73 min). Through analysis of interview transcripts, the following four themes were identified that highlight approaches through which patients in hospitals can be more engaged with laboratory blood testing (Table 2). Quotes are annotated with randomly assigned continuous numbers for each participant (Appendices 4 and 5: Supporting Information: Tables 1 and 2). Themes were common across all participants and no major differences in experiences or recommendations among patient, family member or caregiver participants were noticed. Participants were not asked to review the findings of this manuscript.

**Table 1:** Demographic details of participants.

	Number of participants (*n* = 16)	Percent (%)
Age		
65 years and over	7	44
Under 65 years	8	50
Prefer not to answer	1	6
Gender		
Male	5	31
Female	11	69
Location		
Alberta	9	56
British Columbia	6	38
Both	1^a^	6
Type of hospital		
Urban	11	69
Rural	1	6
Unknown	4	25
Number of comorbidities		
3 or more	8	50
Less than 3	7	44
Prefer not to answer	1	6

^a^ Participant endorsed being admitted to a hospital in each of the provinces in the past 12 months.

**Table 2:** Summary table of approaches to make in‐hospital laboratory testing more patient‐centred according to patient perspectives.

Patient needs	Specific recommendations
Patients need information and education from their healthcare team about expected laboratory blood testing processes as they transition to hospitalization	Develop a specific process of consent that informs patients about laboratory blood testing in hospitals Obtain consent (verbal/written) early during patients' hospitalization, while considering their ability to engage
Laboratory blood draw processes should consider patient comfort and preferences	Discuss patient preferences before blood draws Provide patients with a scheduled time to avoid sleep disruption
Patients prefer to receive explicit information from their healthcare team about the rationale and frequency of laboratory blood testing	Have direct communication with patients and/or family members/caregivers regarding the specific level of detail desired Have a structured discussion that includes rationale for test choice and frequency
Patients need information and education on how laboratory blood testing and results affect their medical care	Discuss test results with patients and/or family members/caregivers Provide an interpretation of how the results guide clinical care

### 3.1 Theme 1: Patients/family members/caregivers need information and education from their healthcare team about expected laboratory blood testing processes as patients transition to hospitalization

Participants recognized the importance of laboratory blood testing in hospitals and acknowledged it to be crucial in helping with diagnosis and monitoring of their state.

I was in there for a serious issue. I thought this [laboratory blood testing] is something that's got to be done because this is how they're going to follow‐up with vital information for my care. (P3, patient)

However, participants indicated that there was a lack of clear explanation at the beginning of patients' hospitalization regarding what they could expect. Most participants had not received direct communication from their healthcare team regarding the rationale and process of laboratory blood testing during their hospitalization.

Doctor provided background information on what the tests would be about and then as a caregiver I sort of sought to find out more information from some reputable online sources as to what the blood tests would actually be for.  (P12, caregiver)

Moreover, none of the participants had explicitly been asked for consent before blood collection. Most were informed about the procedure, so they assumed it was part of their overall hospital care and therefore, provided implied consent by extending out their arm for blood collection.

I've never been asked for consent. It's always the first thing they say is that we're going to draw blood. (P8, patient)

I basically laid there in bed because I wasn't allowed to leave my bed otherwise, I would fall so I was bedridden. And they basically just took my arm out and they did it. (P9, patient)

One caregiver participant indicated that they were aware of the restraints of the healthcare system, and because of this, they avoided asking any questions.

We as a family knew as to how burnt‐out people in the healthcare system are, we were just like ok we're not going to ask for too much…. the issue that crossed my mind was oh well nurses and people in the hospital are all stressed out, burnt out from COVID and such. So we just go with the flow, not just you know avoid asking too many questions. (P12, caregiver)

#### 3.1.1 Patient recommendation

Uniformly, all participants reported that a process of informed consent (verbal or written) was crucial, where they receive information from their healthcare team on the process of blood draws, and why they were needed. One participant suggested that the healthcare team should provide an overview as to what they can expect with the bloodwork process while in the hospital.

…we usually do it at this time every day, sometimes physicians depending on the patient condition need to order it more frequently but here's generally what the routine looks like. Is that going to be ok for you, do we need to make any accommodations and have that conversation… (P2, family member)

The timing of these discussions should consider patients' cognitive or physical limitations.

A conversation upon arriving in the hospital about whether you feel well enough to make decisions about your blood being drawn. (P11, patient)

Yeah, the first few days I don't think I was in the position to be aware and to basically discuss the specifics of the bloodwork but maybe after a week it could have been explained to me what the tests were. (P9, patient)

### 3.2 Theme 2: Laboratory blood draw processes should consider patient comfort and preferences

Participants appreciated when the blood draw process was performed in a gentle, empathetic and accommodating manner. Participants with self‐described ‘poor veins’ had a more difficult and painful experience. Several participants also described extensive bruising attributable to the process. A family member participant was very vocal about their frustration with this process as the person they were caring for was unable to express their pain:

He has very tiny veins that dive and roll anytime they try to get them … it's a very frustrating process because of their lack of success in doing it… He gets poked three times and then the next person comes in and they poke three times. And this just goes on and on. And he becomes a pincushion covered in bruises. (P2, family member)

Many participants were frustrated with blood draws which occurred in the early mornings or late night, leading to sleep disruptions.

The entire process and timing is based on the routines in the hospital. It is not taking into consideration the needs and situation that particular patient is in. And it starts the process off on the wrong foot unfortunately…They flip on the lights particularly in the morning and wake him up when he had just fallen asleep…. (P2, family member)

#### 3.2.1 Patient recommendation

Participants indicated that being considerate of the patient's comfort and eliciting their preferences made laboratory blood testing an engaging process. They appreciated when there had been some prior discussion with them to help them in the process, such as using a butterfly needle or a hot towel to bring out the vein, or the preferred arm to use.

…Leaving the patient, the choice because [they] may have preference on which hand to take the blood sample allows them to participate in the decision‐making process. I think that is important in the sense that they're not being, not manhandled but rather being given a treatment. (P10, patient)

Most participants indicated that they preferred being given a scheduled time for blood draws. All participants preferred blood draws to not happen during early mornings or late nights.

Yeah, make it more in the normal, well to me normal human interaction times. I don't know how many people get up at 6 AM. (P1, patient)

### 3.3 Theme 3: Patients want explicit information from their healthcare team about the rationale and frequency of laboratory blood testing

Most participants reported that laboratory blood testing was not inclusive and expressed frustration with the lack of patient involvement in the process.

I didn't feel like it was some kind of inclusive experience… I felt like I was just there, and they came in and it was hey we're checking your blood. (P3, patient)

We know ourselves best and when there's a chance to know about and discuss what kind of things are affecting our health, I feel it's best to be fully engaged in that. (P1, patient)

Some participants trusted their healthcare team with specific decisions regarding the frequency of testing.

I don't think I was really in a position to get that involved at the time in the first few days. So I think I was operating more on trusting the protocol for the medical practitioner in terms of deciding what my treatment should be and how much information they needed to withdraw in terms of blood drawing. (P9, patient)

Other participants wished for more discussion regarding the frequency of testing and did not understand why such frequency was warranted.

Very frustrating process because of the frequency … does he need them every single day, or does he need them twice a day… (P2, family member)

I have learnt to use my voice…I question everything. You know sure you can do bloodwork every hour but what are you looking for. What's the purpose behind it… (P8, patient)

A caregiver participant revealed that they were not being involved in these types of conversations and the message would be relayed to them by the person they were caring for, who was severely ill:

Well, I can tell you it was not written down. It was not on paper and so if he got anything it probably would have been verbal. And he may just not have shared that exact information with me but even if he did like the verbal pieces, I'm not sure that he would have remembered. (P7, caregiver)

#### 3.3.1 Patient recommendation

While the specific level of detail required varied between participants, all participants preferred being actively involved in their own care, even if this included just receiving information from their healthcare team.

Everybody has autonomy, empowerment and power over their own health and should be involved in absolutely everything to the degree that they can be. (P11, patient)

Participants indicated that creating an environment that gives patients/family members/caregivers opportunities to make decisions is very crucial.

…that effort be made to have the patient and caregiver or family member or whoever it is that the patient wants to be there as part of that care team…the more we can give the patient and caregiver the opportunity to make decisions that they find amenable within the limited parameters in an acute care facility the better we feel about themselves… (P3, patient)

Two participants indicated that written communication and whiteboards are important tools to start this conversation but are often neglected. Most participants indicated that they would have liked to have more information on why their blood was being drawn, especially regarding follow‐up blood tests.

Please tell me what you suspect. If you suspect a urinary tract infection, tell me that. Tell me that's why you are taking the blood test … we're going to have to do it again because we're monitoring the changes… Now you know it's coming; you know what to anticipate. (P2, family member)

### 3.4 Theme 4: Patients need information and education on how laboratory blood testing and results affect their medical care

Most participants reported receiving limited communication regarding laboratory blood test results from the healthcare team during their hospital stay. Participants noted that details of the tests were not communicated to them during their stay unless they asked. Despite being aware of means of accessing test results through online portals, printouts and health records, participants indicated that they were often not in the frame of mind to review their own results during their hospital stay. Several participants indicated that they preferred to discuss the results directly with their healthcare team during hospitalization, and to know how their test results affected their treatment plan.

How would it be related to my treatment plan given my particular disorder. And I think I would have appreciated that if it was offered … this is how it's changed over the past week, and these are our objectives to bring it to this point within the context of that of an appropriate healthy range. (P9, patient)

Here's our interpretation of what this means for you. At this point in time what we want to be doing is another follow up blood test in a day or two or tomorrow morning. (P7, caregiver)

One participant reported that when they received all the information at the end of their hospitalization, they felt burdened by the amount of information shared.

I have so many forms that I got when I was leaving that even I had a bit of information overload, and I haven't looked into it. (P16, patient)

#### 3.4.1 Patient recommendation

Participants indicated that healthcare teams should routinely have conversations with patients to understand their evolving health state. A caregiver participant suggested that including a patient family advisor in the healthcare team could make the process more patient‐centred:

…if there's a patient family advisor on that team, I think it will go a long way to looking at developing a process that supports in a positive way the ability to develop a process that will have great outcomes… (P7, caregiver)

All participants indicated that communication related to tests, test results and rationale for follow‐up tests should be delivered to them by either the attending doctor or the nurse, which would help them understand their results better.

…In terms of actually understanding the results it's so up to I think having a conversation with doctors to best understand. (P12, caregiver)

I'd like to have that explanation plan because just giving me factual information about test results just without any kind of follow‐up doesn't do a lot for me. (P3, patient)

## 4 DISCUSSION

Our study offers insights into current patient experiences with in‐hospital laboratory blood testing and details recommendations from patient participants that can help improve patient engagement with laboratory testing processes in hospitals. Patients indicated that during their hospital stay, they need explicit information and education from their healthcare team on expected laboratory blood testing processes, the rationale and frequency of testing, and how their test results affect their medical care. Patients also expect that the blood testing process should consider their comfort and preferences. The themes identified in our study align with the principles of patient‐centred care, which include respect for patients' preferences and needs, emotional and physical comfort, involvement of family and friends, information and education, and continuity and transition.^34^ This study indicates that in‐hospital laboratory testing processes must become more patient‐centred to allow greater patient engagement. Patient‐centred care results in greater overall satisfaction and improved patient outcomes.^35^, ^36^ As such, patient engagement in the laboratory testing process can encourage partnerships between the patient and their healthcare team. This engagement will improve laboratory test utilization.

Furthermore, the findings of this study are similar to themes identified in prior qualitative studies that capture the patient experience of hospitalization where shared decision‐making and comprehensive communication were central to care.^22^, ^37^, ^38^, ^39^ While patient hospital experience has been studied before, literature regarding patient experiences with laboratory testing, within or outside hospitals, is sparse. Prior literature has shown that patients value testing as part of their medical care,^1^, ^2^, ^3^ appreciate access to their information through inpatient portals^18^, ^19^ and value the relationship with their healthcare team regarding testing decisions.^20^, ^21^ A survey study aiming to understand in‐hospital blood testing from the patient perspective suggested that although patients trust physician ordering practices, they prefer comprehensive communication and value shared decision‐making.^22^ Our study adds rich qualitative detail on how patients ideally envision the interplay of testing decisions, access to test results and their communication with their healthcare team. It also captures patient‐generated recommendations to help make in‐hospital laboratory testing processes more patient‐centred.

Our study is also unique in our engagement of patients as research partners in the team through a PAC, which was guided by the CIHR SPOR—Patient Engagement Framework.^23^ This framework indicates inclusiveness, support, mutual respect and co‐build to be the four guiding principles of patient engagement.^23^ To operationalize these principles, we used several strategies. For instance, patient research partners from diverse backgrounds were recruited, which enabled our reach and access to diverse patient participants. Multiple research‐oriented training workshops were offered so patient research partners could bring their lived‐experience lens to the study's design, data collection and analysis. Compensation was offered for their time and contribution, including additional preparation time. Intentional strategies to foster transparency were used. For instance, co‐development of the Terms of Reference document to indicate a clear description of roles, project timelines, expected time commitments, and so forth. Patient research partners were equal partners in the design of this study, the development of study materials and the conduct of interviews. They reviewed and coded some transcripts contributing to the development of the codebook, along with revising, refining and rewording identified themes, and co‐authoring this manuscript. Formal evaluation of patient engagement within this PAC has also been conducted and the results of this evaluation have been published.^40^ To summarize, patient research partners indicated that support needed to contribute to the project was available and that their opinions were heard; they however suggested that there needs to be more improvement in diversity and collaboration within PAC.^40^ In the future, researchers who are looking to collaborate with patient research partners should understand that meaningful patient engagement requires time and adequate funds, that is, we required an extension in our funding to complete the work. It is important for research teams to factor that into their grant proposals and timelines. Furthermore, meaningful patient engagement requires early involvement to ensure that the study design is relevant for patients, and that right questions are being asked of patient participants.

### 4.1 Implications of research

An important consideration brought forward in this study is the issue of explicit patient consent for tests and treatments in hospitals. While there are explicit consent processes for certain interventions (e.g., transfusion of blood products), there are many other processes (e.g., laboratory testing, imaging investigations, medication changes) that perhaps do not occur with the same form of explicit consent. This study provides an insightful window into the loss of control and autonomy that patients feel as a result of this lack of consent. We propose that hospitalization accompanies an educational orientation to the processes that may occur in hospitals (e.g., laboratory testing, medication changes, etc.), obtain consent for these processes and provide direct opportunities where patients might ask questions around these (e.g., during medical rounds, through their care team, etc.). Our findings also suggest that healthcare providers in hospitals can be more attuned to patients' need for information during acute hospitalization and tailor information sharing according to individual patient preferences.

### 4.2 Limitations

Owing to the COVID‐19 pandemic, we chose convenience sampling for its flexibility and efficiency. A limitation of convenience sampling is motivation bias,^26^ which is a bias that results from being invested in a specific outcome (e.g., a negative blood testing experience). Another weakness of convenience sampling is the risk of limited cases,^26^ of which could have led to limited diversity in our sample given that a majority of the participants identified as females and those who had been admitted in urban hospitals. Similarly, recall bias or a systematic error caused by differences in accuracy of events retrieved from the past^41^ is another limitation to our study as it utilizes interviews to investigate patients’ experiences in hospital (e.g., those with negative experiences in hospital may search their memories more thoroughly than those with positive experiences). Due to COVID‐19 restrictions and limits to travel by the research team, another limitation was that the recruitment poster could only be physically displayed at two hospital sites because of the availability of the research team and feasibility. Additionally, as participant recruitment was predominantly done through technology and posters in the English language, those with limited access to technology or less comfort with the English language could have been missed. This limits the generalizability of our findings to the experiences of all hospitalized patients. However, as evidenced by similar experiences and recommendations of participants within this study, we can say that they are relevant and important to this group of patients and their family members and caregivers and reflect experiences with different hospitals in two Canadian provinces.

## 5 CONCLUSION

In‐hospital laboratory testing processes should become more patient‐centred, enabling opportunities for partnerships with healthcare teams. Healthcare providers should be aware of patient‐identified priorities and encourage patient engagement, which is vital in influencing the appropriate use of laboratory testing in hospitals. Findings from our study can form the basis for the future design of patient‐centred educational tools and processes and engage patients and the health system in improving laboratory test utilization.

## AUTHOR CONTRIBUTIONS

*Substantial contributions to the conception and design of the work; the acquisition, analysis and interpretation of data for the work*: Surakshya Pokharel and Anshula Ambasta. *Substantial contributions to the conception and design of the work, acquisition and analysis*: Zoha Khawaja, Jonathan Williams, Adnan Adil Mithwani, Kimberly Strain, Prachi Khanna, Anna Rychtera and Veronika Kiryanova. *Substantial contributions to the conception of the work, analysis and interpretation of data for the work*: Karen Tang. *Substantial contributions to the design of the work and analysis*: Pamela Mathura and Chris Hylton. In addition to the above, all authors have contributed to the following: Drafting the work or reviewing it critically for important intellectual content; final approval of the version to be published; agreement to be accountable for all aspects of the work in ensuring that questions related to the accuracy or integrity of any part of the work are appropriately investigated and resolved.

## CONFLICT OF INTEREST STATEMENT

The authors declare no conflict of interest.

## ETHICS STATEMENT

The University of Calgary Conjoint Health Research Ethics Board approved this study (Ethics ID no. REB20‐0728).

## Supporting information

Supporting information.

## Data Availability

Original deidentified transcripts are currently securely stored as per institutional policies and are available upon reasonable request in accordance with local ethics board guidelines. All datasets on which the conclusions of the paper rely are within the manuscript and its supporting files.
