# Supplementary material for: Patient perceptions of in‐hospital laboratory blood testing: A patient‐oriented and patient co‐designed qualitative study
Source: Health Expect. 2023 Sep 26;27(1):e13880. doi: 10.1111/hex.13880 (PMC10726148; doi:10.1111/hex.13880)
Supplement: Supplementary file 1 — Supporting information. [file HEX-27-e13880-s001.docx]

**Manuscript:** Patient Perceptions of In-Hospital Laboratory Blood Testing: A Patient-Oriented and Patient Co-Designed Qualitative Study.

**Appendix 1: Consolidated criteria for reporting qualitative studies (COREQ): 32-item checklist.**

Developed from:

Tong A, Sainsbury P, Craig J. Consolidated criteria for reporting qualitative research (COREQ): a 32-item checklist for interviews and focus groups. *International Journal for Quality in Health Care*. 2007. Volume 19, Number 6: pp. 349 – 357

| **No. Item** | **Guide questions/description** | **Reported on Page #** |
| --- | --- | --- |
| **Domain 1: Research team and reﬂexivity** |  |  |
| *Personal Characteristics* |  |  |
| 1. Inter viewer/facilitator | Which author/s conducted the interview or focus group? | Page 6 |
| 2. Credentials | What were the researcher’s credentials? E.g. PhD, MD | Title Page under author credentials |
| 3. Occupation | What was their occupation at the time of the study? | Title Page, Page 6 |
| 4. Gender | Was the researcher male or female? | Page 6 |
| 5. Experience and training | What experience or training did the researcher have? | Page 6 |
| *Relationship with participants* |  |  |
| 6. Relationship established | Was a relationship established prior to study commencement? | Page 6 Inferred as none: “An academic researcher obtained consent from each participant either through email prior to the interview, or during the initial portion of the interview.”  . |
| 7. Participant knowledge of the interviewer | What did the participants know about the researcher? e.g. personal goals, reasons for doing the research | Page 6 |
| 8. Interviewer characteristics | What characteristics were reported about the interviewer/facilitator? e.g. Bias, assumptions, reasons and interests in the research topic | Page 6 |

| **Domain 2: study design** |  |  |
| --- | --- | --- |
| *Theoretical framework* |  |  |
| 9. Methodological orientation and Theory | What methodological orientation was stated to underpin the study? e.g. grounded theory, discourse analysis, ethnography, phenomenology, content analysis | Page 1, 5, 6 |
| *Participant selection* |  |  |
| 10. Sampling | How were participants selected? e.g. purposive, convenience, consecutive, snowball | Page 1, 5, 18 |
| 11. Method of approach | How were participants approached? e.g. face-to-face, telephone, mail, email | Page 1, 5, 6 |
| 12. Sample size | How many participants were in the study? | Page 1, 2, 8, Figure 2 |
| 13. Non-participation | How many people refused to participate or dropped out? Reasons? | Figure 2 |
| *Setting* |  |  |
| 14. Setting of data collection | Where was the data collected? e.g. home, clinic, workplace | Page 1, 6  . |
| 15. Presence of non-participants | Was anyone else present besides the participants and researchers? | Page 6 Inferred as individual interviews |
| 16. Description of sample | What are the important characteristics of the sample? e.g. demographic data, date | Page 1, 5, 8, Table 1, Figure 1 |
| *Data collection* |  |  |
| 17. Interview guide | Were questions, prompts, guides provided by the authors? Was it pilot tested? | Page 6, Appendix Item 2 |
| 18. Repeat interviews | Were repeat interviews carried out? If yes, how many? | No, inferred on page 6, 7 |
| 19. Audio/visual recording | Did the research use audio or visual recording to collect the data? | Page 6 |
| 20. Field notes | Were ﬁeld notes made during and/or after the interview or focus group? | Page 6 |
| 21. Duration | What was the duration of the interviews or focus group? | Page 8 |
| 22. Data saturation | Was data saturation discussed? | Page 1,5 |
| 23. Transcripts returned | Were transcripts returned to participants for comment and/or correction? | Page 6 |
| **Domain 3: analysis and ﬁndings** |  |  |
| *Data analysis* |  |  |
| 24. Number of data coders | How many data coders coded the data? | Page 7 |
| 25. Description of the coding tree | Did authors provide a description of the coding tree? | Page 7, Appendix Item 3 |
| 26. Derivation of themes | Were themes identiﬁed in advance or derived from the data? | Page 7 |
| 27. Software | What software, if applicable, was used to manage the data? | Page 6 |
| 28. Participant checking | Did participants provide feedback on the ﬁndings? | Page 8 |
| *Reporting* |  |  |
| 29. Quotations presented | Were participant quotations presented to illustrate the themes/ﬁndings? Was each quotation identiﬁed? e.g. participant number | Page 8 to 15, Appendix Items 4 and 5 |
| 30. Data and ﬁndings consistent | Was there consistency between the data presented and the ﬁndings? | Yes, there was.  Page 8 to 18 |
| 31. Clarity of major themes | Were major themes clearly presented in the ﬁndings? | Yes. they were.  From page 8 to 15 |
| 32. Clarity of minor themes | Is there a description of diverse cases or discussion of minor themes? | Discussion of themes  From page 15 to 18 |

**Appendix 2:** Interview Guide


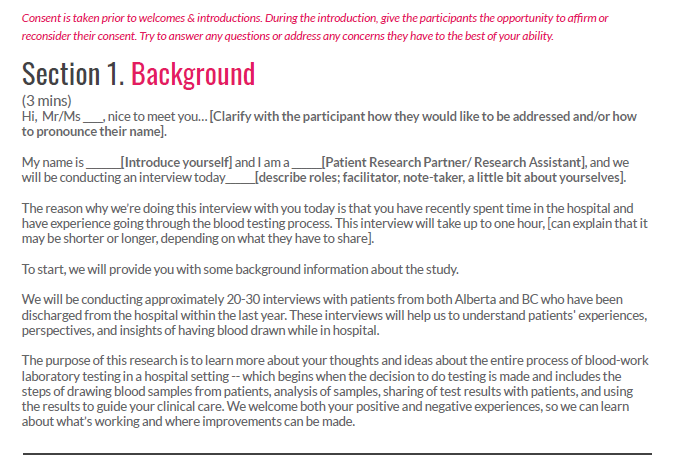


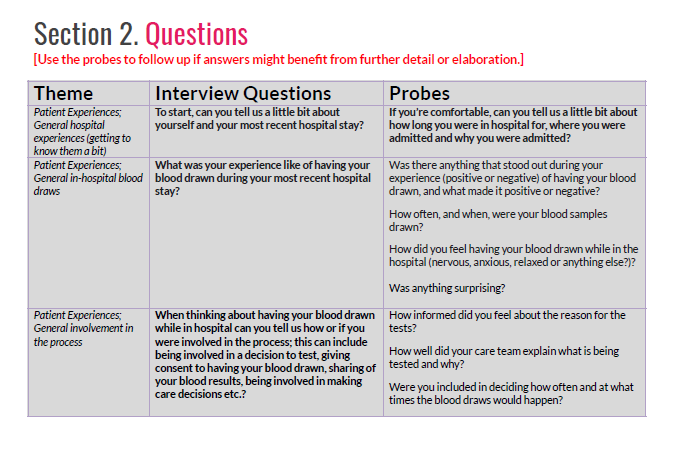

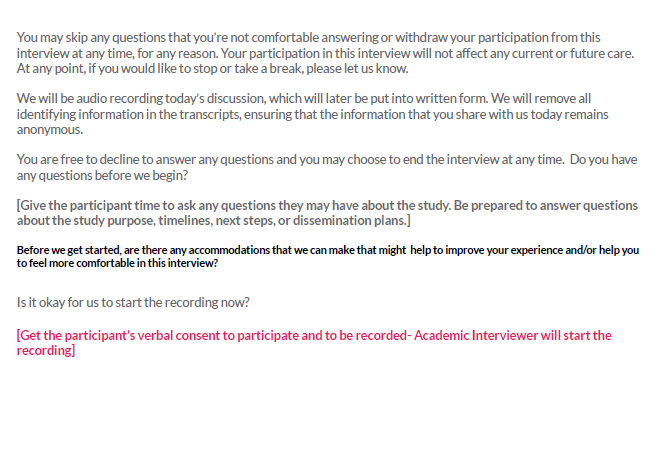


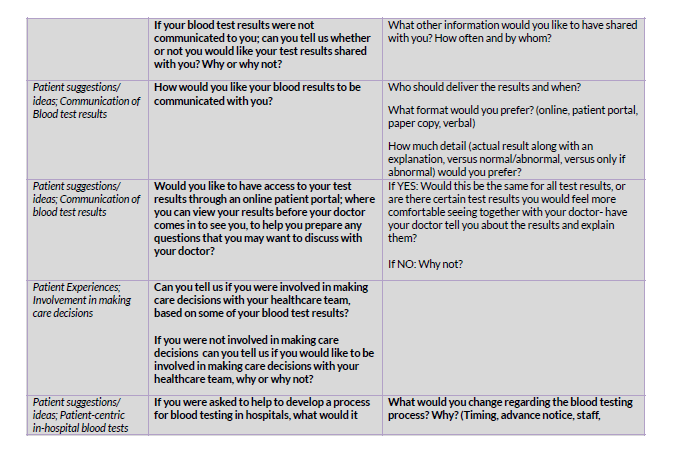

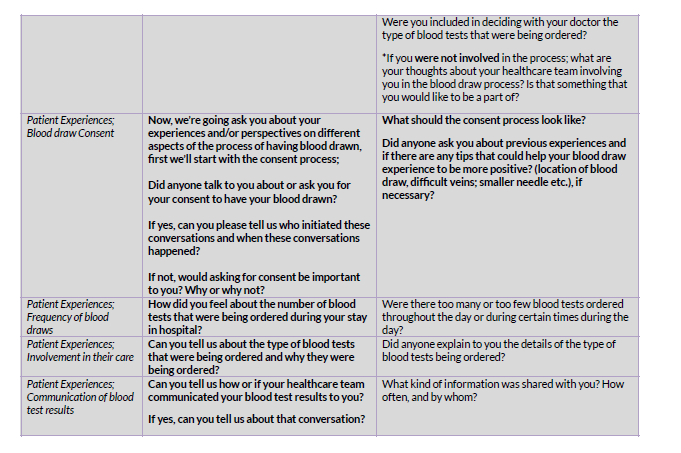


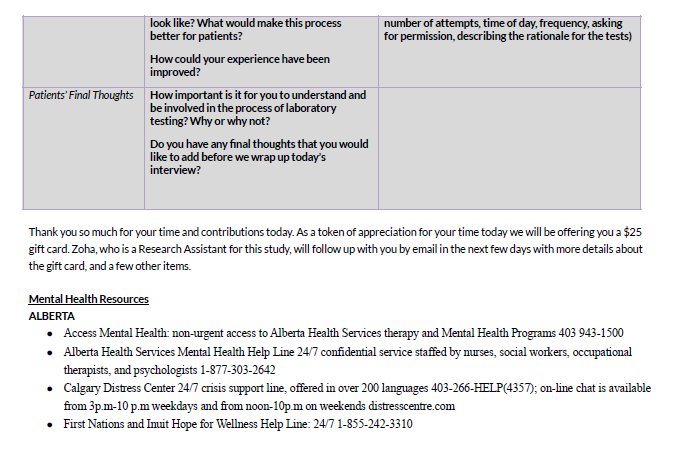


**Appendix 3:** Interview Codebook

**Patient hospital experience:** *Actual incidents that occurred while patient was hospitalized*

- **Blood draws:** *Patient experiences around having their blood drawn*
  - **Consent:** *Any experiences of having or not having consent taken before a blood draw*

*(code neutral experiences/factual information at this level)*

- - - **Positive experiences:** *patient’s positive perspective/experience*
    - **Negative experiences:** *patient’s negative perspective/experience*
  - **Frequency:** *The actual number of blood draws that were done during the patients stay*

*(code neutral experiences/factual information at this level)*

- - - **Positive experiences:** *patient’s positive perspective/experience*
    - **Negative experiences:** *patient’s negative perspective/experience*
  - **Knowledge:** *Any info shared about the blood draw, or any knowledge known about the purpose of the blood draw i.e., why it was being done*

*(code neutral experiences/factual information at this level)*

- - - **Positive experiences:** *patient’s positive perspective/experience*
    - **Negative experiences:** *patient’s negative perspective/experience*
  - **Lab tech:** *Any interactions with the lab tech*

*(code neutral experiences/factual information at this level)*

- - - **Positive experiences:** *patient’s positive perspective/experience/interactions*
    - **Negative experiences:** *patient’s negative perspective/experience/interactions*
  - **Process:** *Whole experience of the process of having their blood drawn*

*(code neutral experiences/factual information at this level)*

- - - **Positive experiences:** *patient’s positive perspective/experience*
    - **Negative experiences:** *patient’s negative perspective/experience*
  - **Timing:** *What time they had their blood drawn*

*(code neutral experiences/factual information at this level)*

- - - **Positive experiences:** *patient’s positive perspective/experience*
    - **Negative experiences:** *patient’s negative perspective/experience*
- **Communication of blood test results:** *How the blood test results were communicated to the patient*
  - **How:** *Was it paper, online, or verbal*

*(code neutral experiences/factual information at this level)*

- - - **Positive experiences:** *patient’s positive perspective/experience*
    - **Negative experiences:** *patient’s negative perspective/experience*
  - **What:** *How much or how little details of the results were shared with them*

*(code neutral experiences/factual information at this level)*

- - - **Positive experiences:** *patient’s positive perspective/experience*
    - **Negative experiences:** *patient’s negative perspective/experience*
  - **When:** *What time was this shared or when did they receive their blood test results*

*(code neutral experiences/factual information at this level)*

- - - **Positive experiences:** *patient’s positive perspective/experience*
    - **Negative experiences:** *patient’s negative perspective/experience*
  - **Who:** *Who communicated these test results to them*

*(code neutral experiences/factual information at this level)*

- - - **Positive experiences:** *patient’s positive perspective/experience*
    - **Negative experiences:** *patient’s negative perspective/experience*
- **Involvement in making care decisions:** *How much or how little were they involved in making their own care decisions. This could be anything from during the time they are having their blood drawn to having their results shared with them as well as their treatment plan afterwards*

*(code neutral experiences/factual information at this level)*

- - - **Positive experiences:** *patient’s positive perspective/experience*
    - **Negative experiences:** *patient’s negative perspective/experience*
- **Other:** *Any other experience shared that doesn’t fall under the above nodes but are important to note e.g., patient history*

**Patient suggestions or recommendations:** *Patient suggestions/recommendations (NOT what actually occurred)*

- **Blood draws:** *Their thoughts on blood draws*
  - **Consent:** *Their thoughts on consent, e.g., what would be considered consent, how the consent process should occur, when should it occur, etc.*
  - **Frequency:** *Their thoughts on how often or how little blood tests should occur i.e., a reduction in the number of blood tests*
  - **Knowledge:** *Their thoughts on how much or how little information should be shared about their blood tests*
  - **Lab tech:** *Their thoughts on lab techs i.e., their roles, how they should approach a patient, how much or little information they should share with the patient, etc.*
  - **Process:** *What they think the process of having their blood drawn should look like*
  - **Timing:** *When they think is a good time to have their blood drawn?*
- **Communication of blood test results:** *How the patients would like the blood test results to be communicated to them*
  - **How:** *How the patient prefers their blood tests to be communication to them i.e., either online, paper, verbal, or all*
  - **What:** *What kind of details they think should be shared e.g., high level summaries, or detailed with explanation*
  - **When:** *When they think is a good time for them to have discussions about their lab test results i.e., should they have access to the results before meeting with the physician, after meeting, or with their family physician, etc.*
  - **Who:** *Who they think should convey the lab test results (nurse, physician, lab tech, etc.)*
- **Involvement in making care decisions**: *How much or how little they would like to be involved in making care decisions*
- **Other:** *Any other topic that was mentioned*

**Appendix 4**: Supplemental Table 1: Themes that Emerged from Interviews with Patients, Family Members, and Caregivers to Describe Patients’ Experience with Blood Testing in Hospitals with Verbatim Quotations

| **Themes** | **Verbatim Quotations** |
| --- | --- |
| 1. Patients/family members/caregivers need information and education from their healthcare team about expected laboratory blood testing processes as patients transition to hospitalization. | “I was in there for a serious issue. I thought this [laboratory blood testing] is something that’s got to be done because this is how they’re going to follow-up with vital information for my care.” (P3, patient)  “Doctor provided background information on what the tests would be about and then as a caregiver I sort of sought to find out more information from some reputable online sources as to what the blood tests would actually be for.” (P12, caregiver)  “I’ve never been asked for consent. It’s always the first thing they say is that we’re going to draw blood…I don’t think I’ve ever been asked. It’s always been told”. (P8, patient)  “I just remember some people just coming in, drawing, saying I’m here to take your bloodwork and basically I just consented or complied” (P3, patient)  “I basically laid there in bed because I wasn’t allowed to leave my bed otherwise, I would fall so I was bedridden. And they basically just took my arm out and they did it.” (P9, patient)  “We as a family knew as to how burnt-out people in the healthcare system are, we were just like ok we’re not going to ask for too much” (P12, caregiver)  “ I think the struggle, not a struggle but the issue that crossed my mind was oh well nurses and people in the hospital are all stressed out, burnt out from COVID and such. So we just go with the flow, not just you know avoid asking too many questions.” (P12, caregiver) |
| 1. Laboratory blood draw processes should consider patient comfort and preferences. | “I would have preferred not to have it done in my feet because I know it gets more painful. But I’ve had to bite the bullet and just because I know my veins are really bad.” (P8, patient)  “Is there any reason to wake me up and take it in the middle of the night?” (P6, patient)  “It was kind of freaky to see my arm was just like blue and orange, whatever because they just hammered it so much by putting stuff in there and taking blood out all the time.” (P3, patient)  “He has very tiny veins that dive and roll anytime they try to get them …it’s a very frustrating process because of their lack of success in doing it…He gets poked three times and then the next person comes in and they poke three times. And this just goes on and on. And he becomes a pincushion covered in bruises.” (P2, family member)  “The whole experience of being in this hospital is very traumatic. So, anything that kind of can sort of lessen that…when you’re in hospital where you just have no control over anything.” (P3, patient)  “The entire process and timing is based on the routines in the hospital. It is not taking into consideration the needs and situation that particular patient is in. And it starts the process off on the wrong foot unfortunately…They flip on the lights particularly in the morning and wake him up when he had just fallen asleep….it doesn’t factor his natural sleep cycles which just further complicates his life when we’re in the hospital…I’ll be like please don’t come in right now, he just fell asleep. He’s hardly slept all night long, please can you come back.” (P2, family member) |
| 1. Patients want explicit information from their healthcare team about the rationale and frequency of laboratory blood testing. | “I didn’t feel like it was some kind of inclusive experience…I felt like I was just there, and they came in and it was hey we’re checking your blood”. (P3, patient)  “He admitted me but he never said is it ok if we take your bloodwork every hour” (P8, patient)  “They did not even explain to why they’re taking the blood samples” (P10, patient)  “You know, I’ve had ten vials of blood taken and nobody has told me what they’re taking them for” (P8, patient)  “… I wanted to know what was going wrong with my body and why I was in hospital. And understand the whole experience more. I like to know what’s going on with my body and I’m like that in everyday life too”. (P15, patient)  “No one really was like front-loading me about hey for next couple of days this is what we’re going to expect so don’t be alarmed” (P 17, patient)  “We know ourselves best and when there’s a chance to know about and discuss what kind of things are affecting our health, I feel it’s best to be fully engaged in that” (P1, patient)  “I felt the whole process was very sort of perfunctory, like I’m lying there, someone comes in and it’s like hey it’s time to take your blood …. then you just have to comply…I checked in on my own volition and now I don’t know when I’m going to get out of here. Like it was just very surreal.” (P3, patient)  “One of the things that really ticks me off is that I’m not involved. They don’t involve me” (P8, patient)  “I don’t think I was really in a position to get that involved at the time in the first few days. So I think I was operating more on trusting the protocol for the medical practitioner in terms of deciding what my treatment should be and how much information they needed to withdraw in terms of blood drawing.” (P9, patient)  “I work in the hospital, and I will chase them away at that time in the morning. I know it’s not critical, I know when it’s critical, when it’s not critical for his care…What made me more vigilant is I’d say anything and hear that this is just routine…and I became a very strong advocate about that because I don’t want wastage in the system….I work in the system…And it’s the frequency of blood draws that increases his risk of stuff. And I know that so it’s for his safety. I’m there for his safety and for his experience.” (P2, family member)  “I trust that they got my best interests in the end. I don’t worry. I’m not a doctor so I don’t really know what they’re doing so I have to trust them” (P4, patient)  “It was not explained to me why it was twice a day…I just assumed that was protocol” (P9, patient)  “I have learnt to use my voice…I question everything. You know sure you can do bloodwork every hour but what are you looking for. What’s the purpose behind it. Why do I need to have this done? Is there something else?” (P8, patient)  “I think that daily seems reasonable to me when somebody’s in the hospital actually with an issue. I mean I’m not sure that they necessarily need it. I guess the way to put it is maybe not everybody needs it but it’s better that everybody gets it every day than somebody gets missed that needed it… I don’t think that, no I don’t think that they overdid it. And they definitely didn’t under do it, that’s for sure. Once a day is about right, I would think.” (P16, patient)  “Very frustrating process because of the frequency…does he need them every single day, or does he need them twice a day (P2, family member)  A caregiver participant indicated that they were not part o  Well I can tell you it was not written down. It was not on paper and so if he got anything it probably would have been verbal. And he may just not have shared that exact information with me but even if he did like the verbal pieces I’m not sure that he would have remembered. (P7, caregiver) |
| 1. Patients need information and education on how laboratory blood testing and results affect their medical care | “It got to the point where I did start saying why are you taking so much, and I never got the results.” (P20, patient)  “If it’s not being looked at… or decisions aren’t being made for every blood draw then why are they being done…just because they are standard routine and they’re scheduled…That’s another frustrating part. Sometimes they [results] are [communicated], most of the time they are not. I have to ask.” (P2, family member)  “How would it be related to my treatment plan given my particular disorder. And I think I would have appreciated that if it was offered…this is how it’s changed over the past week and these are our objectives to bring it to this point within the context of that of an appropriate healthy range.” (P9, patient)  “Here’s our interpretation of what this means for you. At this point in time what we want to be doing is another follow up blood test in a day or two or tomorrow morning.” (P7, caregiver)  “It does not appear from my experience to have been much transparency other than a global statement…there’s an absence of transparency with the patient…Until all the tests are done, and doctor decides to come tell you about it, you’re left with a big question mark.” (P9, patient)  “I didn’t really understand what they were drawing the blood for all the time …it seemed like every day they would change a medication or two which I assume was related to the blood testing.” (P16, patient)  “You can request all your blood tests from your hospital stay so it is available to the patient…it took about a month and a half to get my records.” (P9, patient).  “You have the option of going online and checking your bloodwork…so you know I waited a bit and went online…I have an app on my phone so I can look at my results.” (P8, patient)  “I have so many forms that I got when I was leaving that even I had a bit of information overload, and I haven’t looked into it.” (P16, patient) |

**Appendix 5:** Supplemental Table 2: Mapping Patients, Family Members, and Caregivers’ Recommendations to the Four Emerged Themes for Improving the Process of Blood Testing in Hospitals with Verbatim Quotations

| **Theme** | **Recommendation** | **Verbatim** **Quotations** |
| --- | --- | --- |
| 1. Patients/family members/caregivers need information and education from their healthcare team about expected laboratory blood testing processes as patients transition to hospitalization. | Provide patients information on consent, an overview of what to expect and be considerate of their limitations from their hospitalization. | “Look I know you have a job to do. You have to do this, but I do appreciate the fact that you’re asking me in case I have any legitimate concerns about this process…. it’s like it’s one thing to ask consent but if you’re also giving maybe a little 30 second to 1 minute preamble as to like what’s behind doing this and what the reasoning for this. And how that’s going to benefit my healthcare.” (P3, patient)  “Have a conversation upon arriving in the hospital about whether you feel well enough to make decisions about your blood being drawn….Have a conversation with the physician or the registered nurse in [their] care and make the decision based on that. And [patient] not being completely at their best I think it’s important to cover all base.” (P11, patient)  “I would love to have somebody take two minutes to explain to me what they’re doing. And yeah, it’s just bloodwork but what is it. What are you taking and why are you taking it… “They don’t tell you they’re doing it. You have to ask so it would be nice just for them to say we like to do a basic or we’d like to do something a little bit more.” (P8, patient)  “I think something right from the beginning, say here’s what our blood routine is.... We usually do it at this time every day, sometimes physicians depending on the patient condition needs to order it more frequently but here’s generally what the routine looks like. Is that going to be ok for you, do we need to make any accommodations and have that conversation. That would be an amazing first step.” (P2, family member)  “I would have appreciated it being volunteered to me without me having to specifically ask, given the state of mind I was in certainly in the first week.” (P9, patient)  “I think there should be a degree of judgement from the physician just because I wasn’t terribly well at the time about when to approach me with that conversation...”(P11, patient)  “Yeah the first few days I don’t think I was in the position to be aware and to basically discuss the specifics of the bloodwork but maybe after a week it could have been explained to me what the tests were.” (P9, patient) |
| 2. Laboratory blood draw processes should consider patient comfort and preferences. | Examples of preferences and comfort include asking patients their preferred arm for venipuncture, providing a scheduled time for blood draws that are not early mornings or late nights etc. | “…when a lab draw is being made having to inform the person all of the time don’t use this arm, use this arm. You know like it would just kind of let you know that oh ok they remembered. Right and yeah, I think that would be a very supportive action.” (P7, caregiver)  “Leaving the patient the choice because [they] may have preference on which hand to take the blood sample allows them to participate in the decision-making process. I think that is important in the sense that they’re not being, not manhandled but rather being given a treatment.” (P10, patient)  “Yeah make it more in the normal, well to me normal human interaction times. I don’t know how many people get up at 6 AM.” (P1, patient)  “it was either very late at night or very early in the morning so it’s like other than time frame it’s still all good.” (P14, patient)  “even better if it can be complimented by that conversation with doctor or nurse and having a paper copy.” (P12, caregiver)  “…considering I was in the hospital it would be difficult to approach an online format just because sometimes they’re not compatible with your phone…everybody learns differently and everybody might be curious in different ways about the level of detail that they could obtain from like an online record versus just a general conversation.” (P11, patient)  “This is a person’s bedroom, there’s people sleeping in here. Yeah, I would like to see a bit more understanding or a sign on the door that says there’s a family member who’s staying 24/7… or this patient has been up all night, please you know I’d love the nursing staff to be able to warn the labs this patient shouldn’t be woken at six in the morning.” (P2, family member)  “In truth, in terms of taking blood and the testing part of it they do the best they can and a lot of it is procedure. I realize that. They need a little bit of, so does the doctor, bedside manner training just so the interpersonal relationships are a little stronger…I would like to see a little bit more interaction between the nursing, well interpersonal reaction between the nursing and the patient.” (P6, patient) |
| 3. Patients want explicit information from their healthcare team about the rationale and frequency of laboratory blood testing. | - Provide patients and their families /caregivers opportunities to be actively involved in their care  - use simple language to communicate | “everybody has autonomy, empowerment and power over their own health and should be involved in absolutely everything to the degree that they can be.”(P11, patient)  “...just asking the patient what you want… when do you want and how do you want the lab information given to you … maybe let’s help sort out that care plan.” (P7, caregiver)  “I think it’s even more important … that there’s that effort made to have the patient and caregiver or family member or whoever it is that the patient wants to be there as part of that care team…the more we can give the patient and caregiver the opportunity to make decisions that they find amenable within the limited parameters in an acute care facility the better we feel about themselves.”(P3, patient)  “I wouldn’t need them to go into detail because I probably wouldn’t even understand. So keeping it basic, simple.” (P5, patient)  “It’s nice to have them say everything is normal but then if they say ok it appears there may be something abnormal in this area then I think I need to know that.” (P10, patient)  “Could also just be normal and abnormal…” (P4, patient)  “Please tell me what you suspect. If you suspect a urinary tract infection tell me that. Tell me that’s why you are taking the blood test…we’re going to have to do it again because we’re monitoring the changes... Now you know it’s coming, you know what to anticipate.”(P2, family member)  “It’s always you know we’ve taken your bloodwork and I would love it to have somebody say to me this is what it’s showing but we aren’t concerned about it or we need to do something about it or whatever.” (P8, patient)  “It almost would be nice if the doctor and whoever is in charge of the nursing effort met with you fairly early into it to say this is what we think you have, and this is what we’re test for to find out.”(P4, patient) |
| 4.Patients need information and education on how laboratory blood testing and results affect their medical care. | - Have routine one-on-one conversations with patients  - Doctor or nurse should deliver communication related to tests, results, treatment plans | “…in terms of actually understanding the results it’s so up to I think having a conversation with doctors to best understand.” (P12, caregiver)  “That somebody would explain to me what the results are and what it means.”(P15, patient)  “…I’d like to have that explanation plan because just giving me factual information about test results just without any kind of follow-up doesn’t do a lot for me.” (P3, patient)  “So just to know that if everything’s ok or something is wrong then I would like to know rather than just guessing that I guess everything’s ok.” (P5, patient)  “Even if it’s fine. If they’ve taken my blood and there’s no infection. There’s no issue, nice to know.” (P6, patient)  “…if there’s a patient family advisor on that team, I think it will go a long way to looking at developing a process that supports in a positive way the ability to develop a process that will have great outcomes. And where patients and families can feel involved in the long run.”. (P7, caregiver) |
